# A Comparative Study of Seroprevalence of 17 Common Pathogens Among Airline Pilots and Office Workers

**DOI:** 10.7759/cureus.50778

**Published:** 2023-12-19

**Authors:** Andrés Santiago Sáez, Ángel García Martín, Manuel Gómez Serrano, Miryam Liaño Riera, Piercarlo Minoretti

**Affiliations:** 1 Legal Medicine, Hospital Clinico San Carlos, Madrid, ESP; 2 Legal Medicine, Psychiatry, and Pathology, Complutense University of Madrid, Madrid, ESP; 3 General Direction, Studio Minoretti, Oggiono, ITA

**Keywords:** airline pilots, occupational medicine, infections, seroprevalence, office workers

## Abstract

Background

The variation in infection risk among individuals is thought to be partially influenced by occupational factors. This study aims to investigate the seropositivity rates of 17 common infectious agents in male airline pilots (APs), a professional group known to experience a high prevalence of cardiovascular and gastrointestinal diseases.

Methodology

In our study, we employed a case-control design with 100 male APs as cases, matched by age, sex, and tenure (i.e., at least five years of service) to 100 male office workers (OWs) who served as controls. We measured the IgG antibody levels to 17 pathogens using specific enzyme-linked immunosorbent assays, including herpes simplex virus 1, herpes simplex virus 2, varicella-zoster virus, Epstein-Barr virus, cytomegalovirus, human herpesvirus 6, human herpesvirus 7, Kaposi’s sarcoma-associated herpesvirus, *Toxoplasma gondii*, human T-lymphotropic virus 1, BK virus, John Cunningham virus, Merkel cell polyomavirus, human papillomavirus 16, human papillomavirus 18, *Chlamydia trachomatis*, and *Helicobacter pylori*. The determination of seropositivity cutoffs for each pathogen was made in accordance with the guidelines provided by the respective kit manufacturers.

Results

The seropositivity rates for the 17 pathogens ranged from 1% for human T-lymphotropic virus 1 to 94% for varicella-zoster virus and were similar in both professions, except for herpes simplex virus 1 and *Helicobacter pylori*, which were more prevalent in APs.

Conclusions

Our findings suggest a higher prevalence of previous infections with herpes simplex virus 1 and *Helicobacter pylori *in APs compared to OWs. These infections may be associated with the prevalence of specific non-communicable diseases in this professional group. However, additional longitudinal studies are needed to substantiate this hypothesis.

## Introduction

Persistent infections caused by various pathogens, including viruses, bacteria, and parasites, have been identified as a contributing factor to the development of non-communicable diseases (NCDs) such as cardiovascular disease (CVD) and cancer in the general population [1]. In addition, research indicates a correlation between persistent pathogen-induced inflammation and the progression of atherosclerotic vascular disease [2]. The concept of pathogen burden, which refers to the cumulative exposure to different infectious agents [3], has emerged as a potential determinant of NCD risk. Seropositivity, determined through IgM and/or IgG antibody testing, is commonly used to assess infection prevalence within a community [4]. Interestingly, various factors including education levels, socioeconomic status, and occupational factors have been found to influence seropositivity rates [3,5]. These rates depict the proportion of individuals who test positive for specific pathogen antibodies relative to the total number of individuals tested.

Airline pilots (APs) encounter various occupational hazards that can have adverse effects on their health and well-being [6]. The nature of their work, which involves irregular flight schedules, shift work, and travel across different time zones, often disrupts their natural body rhythms. In addition, pilots may suffer from fatigue, exposure to cosmic radiation, irregular meal times, disrupted sleep patterns, and even symptoms of depression [6-10]. The sedentary nature of their job and the cabin environment, characterized by noise and vibrations [11], further compound these challenges. Previous studies have indicated a higher prevalence of malignant melanoma [12] among pilots compared to the general population. Additionally, this professional group exhibits a significant incidence of cardiometabolic risk factors [8] and functional gastrointestinal disorders (FGIDs) [13]. However, the potential impact of infectious pathogens on exacerbating these health issues remains uncertain due to the lack of seroepidemiological investigations specifically focused on common infectious pathogens among APs. Consequently, there is a dearth of data on seropositivity rates for viruses, bacteria, and parasites that may be associated with NCDs within this particular population.

In this study, we conducted an extensive analysis of sera collected from APs to determine the rates of seropositivity (IgG antibodies) for 17 pathogens, including herpes simplex virus 1 (HSV-1), herpes simplex virus 2 (HSV-2), varicella-zoster virus (VZV), Epstein-Barr virus (EBV), cytomegalovirus (CMV), human herpesvirus 6 (HHV-6), human herpesvirus 7 (HHV-7), Kaposi’s sarcoma-associated herpesvirus (KSHV), *Toxoplasma gondii *(*T. gondii*), human T-lymphotropic virus 1 (HTLV-1), BK virus (BKV), John Cunningham virus (JCV), Merkel cell polyomavirus (MCV), human papillomavirus 16 (HPV-16), human papillomavirus 18 (HPV-18), *Chlamydia trachomatis *(*C. trachomatis*), and *Helicobacter pylori *(*H. pylori*). While most infections caused by these pathogens generally result in mild or subclinical symptoms, there are cases where they can lead to severe manifestations and, more importantly, increase the risk of NCDs. To compare the findings, we selected a control group of office workers (OWs) who were matched for age, sex, and tenure (i.e., at least five years of service).

## Materials and methods

Participants

In our study, we employed a case-control design with 100 male APs as cases, matched by age, sex, and tenure (i.e., at least five years of service) to 100 male OWs who served as controls. The subjects were voluntarily recruited during routine occupational health assessments at outpatient clinics, with invitations to participate disseminated by an occupational health physician. To ensure accurate results, we matched each OW with an AP in terms of age, sex, and tenure, with a minimum requirement of five years of service. Office professionals who were subjected to shift work, extensive travel, or disruptions to their circadian rhythm were not eligible for inclusion. We excluded women due to their limited representation within the AP population. Participants with a history of diabetes, CVDs, malignancies, or recent drug therapy were also excluded. Additionally, none of the participants were using dietary supplements, and they all appeared to be in good physical health. All procedures were conducted at outpatient facilities owned by Studio Minoretti Srl (Oggiono, Italy). The research adhered to the ethical standards outlined by the Declaration of Helsinki and received approval from the local ethics committee (Studio Minoretti; reference number: 2021/12SES). We obtained written informed consent from each participant before including them in the study.

Serological testing and definitions

Participants provided a fasting venous blood sample of 10 mL, which was collected into serum collection tubes. The sera were then analyzed using commercially available enzyme-linked immunosorbent assay antibody test kits to detect IgG antibodies against 17 common pathogens (HSV-1, HSV-2, VZV, EBV, CMV, HHV-6, HHV-7, KSHV, *T. gondii*, HTLV-1, BKV, JCV, MCV, HPV-16, HPV-18, *C. trachomatis*, and *H. pylori*). The manufacturers’ protocols were followed to determine the seropositivity cutoffs for each pathogen, and the results were reported as either positive or negative. Out of the 17 pathogens, five (EBV, HTLV-1, *H. pylori*, HPV-16, and HPV-18) are classified as group I human carcinogens by the International Agency for Research on Cancer [3]. The pathogen burden was assessed based on the percentage of individuals within each professional group who tested positive for more than 10 infectious pathogens [3] and more than two oncogenic pathogens [3].

Statistical analysis

The normality of continuous data was assessed using the Kolmogorov-Smirnov test, which revealed that all variables followed a normal distribution. This confirms the appropriateness of using parametric statistical methods. Continuous variables were presented as mean ± standard deviation (SD), while categorical data were expressed as counts and percentages. To compare the two professions, the Student’s t-test was used for continuous data, and the chi-squared test was applied for categorical variables, including seropositivity counts. Crude odds ratios (ORs) were calculated to describe the associations between the seropositivity status and each professional category. The statistical analyses were conducted using SPSS software version 20.0 (IBM Corp., Armonk, NY, USA), and all tests were two-sided with a significance level of 5%.

## Results

Table 1 provides an overview of the key characteristics observed among the participants.

**Table 1:** General characteristics of the study participants. Data are expressed as means ± standard deviations unless otherwise indicated. ns: not significant

Variable	Airline pilots (n = 100)	Office workers (n = 100)	P-value
Men	100 (100%)	100 (100%)	ns
Age, years	39.8 ± 4.7	40.3 ± 5.0	ns
Length of service, years	10.1 ± 4.2	10.4 ± 4.5	ns
Current smoking, n (%)	18 (18.0%)	21 (21.0%)	ns
Body mass index, kg/m^2^	24.7 ± 2.9	25.1 ± 3.1	ns
Total cholesterol, mg/dL	216 ± 15	224 ± 19	ns
Fasting plasma glucose, mg/dL	94 ± 11	96 ± 12	ns

The two groups did not differ significantly in various aspects, including age, service duration, current smoking habits, body mass index, total cholesterol, and fasting plasma glucose levels. Table 2 provides an overview of the seropositivity rates for each pathogen, with percentages ranging from 1% for HTLV-1 to 94% for VZV.

**Table 2:** Seropositivity for 17 different pathogens in airline pilots and office workers. Pathogens classified as group I human carcinogens by the International Agency for Research on Cancer are marked in bold. ns: not significant; HSV-1: herpes simplex virus 1; HSV-2: herpes simplex virus 2; VZV: varicella-zoster virus; EBV: Epstein-Barr virus; CMV: cytomegalovirus; HHV-6: human herpesvirus 6; HHV-7: human herpesvirus 7; KSHV: Kaposi’s sarcoma-associated herpesvirus; *T. gondii*: *Toxoplasma gondii*; HTLV-1: human T-lymphotropic virus 1; BKV: BK virus; JCV: JC virus; MCV: Merkel cell polyomavirus; HPV-16: human papillomavirus type 16; HPV-18: human papillomavirus type 18; *C. trachomatis*: *Chlamydia trachomatis*; *H. pylori*: *Helicobacter pylori*

Positive IgG serology, n (%)	Airline pilots (n = 100)	Office workers (n = 100)	P-value
HSV-1	78 (78%)	60 (60%)	0.006
HSV-2	12 (12%)	10 (10%)	ns
VZV	94 (94%)	92 (92%)	ns
EBV	78 (78%)	74 (74%)	ns
CMV	61 (61%)	57 (57%)	ns
HHV-6	77 (77%)	81 (81%)	ns
HHV-7	90 (90%)	87 (87%)	ns
KSHV	15 (15%)	11 (11%)	ns
T. gondii	21 (21%)	16 (16%)	ns
HTLV-1	2 (2%)	1 (%)	ns
BKV	88 (88%)	90 (90%)	ns
JCV	61 (61%)	56 (56%)	ns
MCV	58 (58%)	60 (60%)	ns
HPV-16	5 (5%)	3 (3%)	ns
HPV-18	3 (3%)	2 (2%)	ns
C. trachomatis	19 (19%)	16 (16%)	ns
H. pylori	38 (38%)	21 (21%)	0.009

The prevalence rates were comparable between both professions, with the exception of HSV-1 and *H. pylori*, which were more common among APs. The crude ORs showed that APs were 2.35 times more likely to be seropositive for HSV-1 (95% confidence interval (CI) = 1.27-4.39, p = 0.006) and 2.30 times more likely to be seropositive for *H. pylori* (95% CI = 1.23-4.32, P = 0.009) compared to OWs.

All participants tested positive for at least one pathogen. Among them, 23 APs (23%) and 13 OWs (13%) exhibited seropositivity for over 10 pathogens, suggesting a potentially higher pathogen burden in APs. However, this difference did not reach statistical significance (crude OR = 1.83, 95% CI = 0.88-3.81, p = 0.10). A similar non-significant trend was observed for seropositivity for more than two oncogenic pathogens. Specifically, nine APs (9%) and three OWs (3%) had this condition, resulting in a crude OR of 3.16 (95% CI = 0.83-12.04, p = 0.09).

## Discussion

This study is the first to compare the seropositivity rates of 17 common infectious agents in two distinct groups: APs, a professional category commonly affected by NCDs [6,8], and OWs. Additionally, it provides valuable insights into specific oncogenic pathogens. The study revealed three key findings. First, APs exhibited higher prevalence rates of HSV-1 and *H. pylori *compared to OWs. Second, although not statistically significant, there was a tendency toward a higher pathogen burden in APs, defined as seropositivity for more than 10 pathogens. Lastly, a similar trend was observed for the seroprevalence of more than two oncogenic pathogens.

Human infection with HSV-1, a member of the herpesviridae family, poses a significant public health concern [14]. In 2016, global estimates indicated that approximately 3.752 billion individuals were infected with HSV-1, accounting for a worldwide incidence of 66.6% in the age group between 0 and 49 years [15]. Our study findings align with this epidemiological trend, revealing a seroprevalence of 78% among APs and 60% among OWs. Primary infection with HSV-1 is usually asymptomatic, but in rare cases, it can result in severe encephalitis [16]. Following the primary infection, the virus enters a latent phase where it remains inactive within neurons. Reactivation of the virus is usually triggered by stress-related immune system dysregulation and can manifest as grouped vesicular papules with inflammatory components on the oral, corneal, and/or genital mucocutaneous surfaces [16]. Additionally, the host’s immune response, specifically cytokine-mediated immunologic reactions, can also contribute to the development of systemic diseases associated with HSV-1 [16]. Intriguingly, studies have demonstrated a potential association between the seroprevalence of anti-HSV-1 antibodies and CVD [17-19]. Remarkably, the burden of cardiovascular risk factors appears to be significantly elevated among APs [7,8,20]. In a cohort study conducted by Siscovick et al. [17] in the United States, it was discovered that older individuals with IgG antibodies against HSV-1 were twice as likely to experience myocardial infarction and cardiovascular mortality. Similarly, Jafarzadeh et al. [18] found that Iranian patients with ischemic heart disease had significantly higher anti-HSV-1 antibody seroprevalence compared to a healthy control group. Additionally, a meta-analysis by Wu et al. [19] of 17 studies revealed a 1.77-fold increased risk of atherosclerosis in patients infected with HSV-1. Despite these findings, the role of HSV-1 in CVD remains a matter of debate due to inconsistent results in the published literature [21]. It is also worth noting that gastric HSV-1 infection has also been linked to FGIDs [22], which have been previously reported in APs [13]. Unlike space flight, where HSV-1 infections have been more thoroughly investigated [23,24], the reasons behind the higher HSV-1 seroprevalence in APs compared to OWs are currently speculative as this finding is reported for the first time. However, pilots’ immune system dysregulation due to high fatigue and circadian disruption [6,10] as well as factors related to local air recirculation in current airliner cabins [11] may represent plausible explanations. Although acute HSV-1 infections do not affect pilots’ fitness to fly, medical advice should be given regarding the shedding of viral DNA from saliva and oral secretions.

*H. pylori*, a gram-negative and spiral-shaped bacterium that predominantly colonizes and proliferates within the gastric mucosa, has been linked to both peptic ulcer disease [25] and FGIDs [26]. In our study, we observed a significantly higher prevalence of* H. pylori* IgG antibodies in APs compared to OWs. Regulatory authorities have established guidelines for aeromedical examiners, stipulating that pilots with stable and effectively managed peptic disease may be granted fitness-to-fly certifications. Nonetheless, individuals who have experienced an active ulcer must demonstrate a minimum of three months of stability, devoid of symptoms. In cases where the ulcer has resulted in bleeding, a six-month period of stability is required. Hence, *H. pylori* infection could potentially be an overlooked factor contributing to significant loss of work hours in APs. Moreover, individuals infected with *H. pylori* may exhibit FGIDs, particularly if they also experience dysbiosis of gut microbiota [27]. A study involving 212 male pilots from a prominent Chinese airline revealed that an estimated 39% suffered from FGIDs [13], a figure strikingly similar to the *H. pylori* seropositivity rate observed in our study (38%). In a recent investigation, we have also highlighted a notable reduction in health-promoting bacterial species within the gut microbiota of APs [28]. This elevated *H. pylori* infection rate, in conjunction with intestinal dysbiosis [28], could potentially account for the reported high incidence of FGIDs within this occupational group. Furthermore, *H. pylori *infection has been linked to various extraintestinal manifestations, such as CVD, obstructive sleep apnea syndrome, and metabolic syndrome [29]. These are conditions that have been previously reported in the pilot population [6-8], adding to the significance of this association. Another intriguing observation is the connection between *H. pylori* and motion sickness. A retrospective study conducted among pilot trainees undergoing basic flight training revealed that those who tested positive for *H. pylori *and received eradication therapy experienced a temporary reduction in reported nausea during flight training [30]. Although the exact transmission routes of *H. pylori* are not fully understood, it is hypothesized that overcrowding may serve as a transmission risk factor [31]. Consequently, highly occupied spaces such as aircraft cabins [11] can make APs more susceptible to contracting the infection. In addition, the recirculation of air within the cabin [11] could potentially contribute to this risk. This factor could also explain the non-significant trends observed toward a higher pathogen burden, including seropositivity for over 10 pathogens, as well as seropositivity for more than two oncogenic pathogens in APs compared to OWs.

Our study boasts a notable strength in its thorough evaluation of IgG antibodies across various pathogens. Furthermore, we made efforts to carefully match both occupational groups in terms of general characteristics, effectively minimizing the influence of confounding variables on the seropositivity rates. However, it is essential to acknowledge the limitations of our research. The relatively small sample size introduces an element of uncertainty, preventing us from drawing definitive conclusions. Furthermore, the voluntary participation of APs and OWs may introduce self-selection bias, limiting the generalizability of our findings. The exclusion of female participants also hinders the applicability of our results to women. Additionally, we did not collect data on potential determinants or correlates for infections, which are typically not gathered during occupational health visits. Lastly, the absence of endoscopy data prevents us from establishing a correlation between *H. pylori *seropositivity and the presence of peptic ulcer disease.

## Conclusions

Our research indicates a significantly higher prevalence of prior infections with HSV-1 and *H. pylori* among APs compared to OWs. These infections could potentially be linked to the high prevalence of certain NCDs observed within this professional group. However, these findings are preliminary and should be interpreted with caution. To further validate this hypothesis and establish a more definitive causal relationship, it is crucial to conduct additional longitudinal studies. These future investigations will provide a more comprehensive understanding of the occupational health risks associated with these infections and inform appropriate preventative measures.
